# Lifetime history of concussion among children and adolescents with attention-deficit/hyperactivity disorder: examining differences stratified by age, medication status, and parent-reported severity

**DOI:** 10.3389/fneur.2024.1487909

**Published:** 2025-01-29

**Authors:** Julia E. Maietta, Grant L. Iverson, Nathan E. Cook

**Affiliations:** ^1^Department of Physical Medicine and Rehabilitation, Harvard Medical School, Boston, MA, United States; ^2^Department of Physical Medicine and Rehabilitation, Spaulding Rehabilitation Hospital, Charlestown, MA, United States; ^3^Mass General for Children Sports Concussion Program, Waltham, MA, United States; ^4^Schoen Adams Research Institute at Spaulding Rehabilitation, Cambridge, MA, United States; ^5^Department of Psychology, University of Rhode Island, Kingston, RI, United States

**Keywords:** attention-deficit/hyperactivity disorder, concussion, traumatic brain injury, ADHD severity, ADHD medication

## Abstract

**Introduction:**

Children and adolescents with attention-deficit/hyperactivity disorder (ADHD) are more likely to report a lifetime history of concussion compared to those without ADHD. Prior studies have predominantly relied on surveys of youth who self-reported their ADHD status (as opposed to parent report), examined samples with relatively narrow age ranges, and focused on youth athletes. It is unclear if ADHD severity or ADHD medication influences the association between ADHD and greater lifetime history of concussion. We investigated the association between parent-reported ADHD, ADHD severity, and medication status with parent-reported lifetime concussion history in children and adolescents ranging in age from 5 to 17, sampled from the United States general population.

**Methods:**

Parent reported health data from 33,914 children and adolescents were selected from the 2021 National Survey of Children’s Health. Lifetime history of concussion, current ADHD diagnosed by a healthcare provider, and, for those with current ADHD, ADHD severity (mild or moderate/severe), and current ADHD medication status (yes/no) were reported by parents. A Mantel–Haenszel analysis was used to assess the associations between ADHD and lifetime concussion history across five age groups (ages 5–7, 8–10, 11–13, 14–15, and 16–17). Additional Mantel–Haenszel analyses were used to assess the associations between lifetime history of concussion and ADHD severity as well as medication status across five age groups.

**Results:**

Overall, 6.2% of youth had a parent-reported history of concussion and 12.3% had parent-reported current ADHD. Twice as many youth with ADHD (10.6%) had a parent-reported history of concussion compared to youth without ADHD (5.6%). There were no differences in lifetime concussion history for those with mild (10.9%) versus moderate/severe ADHD (10.3%). Similarly, among those with ADHD, there were no differences in lifetime concussion history for those taking medication (9.9%) versus those not taking medication (11.6%).

**Discussion:**

Consistent with previous literature, youth with ADHD had greater lifetime concussion history than youth without ADHD (approximately twice the prevalence in those with ADHD). Contrary to our hypotheses among youth with ADHD, parent-reported ADHD severity and current medication status were not associated with lifetime concussion history.

## 1 Introduction

Attention-deficit/hyperactivity disorder (ADHD) is a neurodevelopmental disorder characterized by symptoms such as inattention, hyperactivity, and impulsivity (1). The condition can be characterized further based on severity as either mild, moderate, or severe according to the number of symptoms and level of functional impairment (1). Meta-analyses have found that individuals with ADHD have 1.5 to 2 times the rate of overall bodily injuries compared to those without ADHD (2, 3). Numerous studies have examined the incidence and lifetime prevalence of concussion among individuals with and without ADHD. Studies consistently find that individuals with ADHD are more likely to have a history of concussion compared to individuals without ADHD (4–11). Of note, studies have generally not found gender differences; that is, rates of prior concussion appear similar between boys with ADHD and girls with ADHD (5, 8, 12). The precise reasons for this association and increased risk for injury and concussion remain unclear. Youth with ADHD have been found to have lower scores on tests of balance and postural stability (13–16), and difficulties with balance as well as higher rates of hyperactivity/impulsivity have been theorized to contribute to higher self-reported concussion history and history of other orthopedic injuries in ADHD populations (13, 14). However, available studies have examined samples with relatively narrow age ranges that center predominantly around adolescents (see Table 1). It is unclear whether the well-established increased rates of prior concussions among those with ADHD is consistent at different ages.

**Table 1:** Studies examining history of concussion among those with ADHD (organized by average sample age, from youngest to oldest).

Author	Ages (Mean)	Sample size	ADHD sample size	Sample recruitment context	How ADHD was determined	Concussion history		
						ADHD	No ADHD	*p*
Liou et al. (2018) (33)	3–29 (9.3)	144,362	71,181	National database in Taiwan	Diagnosis code (from medical record review)	4.3%	1.0%	<0.001
Cook, Karr, et al. (2022) (12)	9–10 (9.9)	10,585	1,085	U.S. population cohort	Parent report	7.2%^a^	3.2%^a^	<0.001
Iverson et al. (2021) (8)	11–14 (12.6)	1,037	71	MS student athletes	Self-report	23.9%^b^	11.4%^b^	0.002
Terry et al. (2020) (35)	12–13 (NR)	1,744	77	MS student athletes	Self-report	16.9%	13.6%	0.41
Cook, Teel, et al. (2022) (5)	5–18 (13.6)	2,418	294	Concussion clinic	Self/parent-report	43.9%^c^	37.5%^c^	0.04
Hrabarchuk et al. (2024) (36)	12–22 (14.9)	7,196	276	Student athletes	Self-report	8.7%^d^	5.8%^d^	0.04
Iverson et al. (2020) (7)	13–18 (15.5)	32,487	1,324	HS student athletes	Self-report	24.5%	16.1%	<0.001
Mautner et al. (2015) (37)	13–18 (15.6)^e^	140	70	MS & HS student athletes	Self-report	41.4%	25.7%	.11^f^
Salinas et al. (2016) (38)	12–18 (15.6)^e^	512	256	HS student athletes	Self/parent-report	64.0%	36.0%	0.02
McLeod et al. (2018) (11)	NR (15.7)	8,814	375	HS student athletes	Self-report	27.0%^g^	17.0%^g^	<0.001
Iverson et al. (2016) (6)	13–19 (15.9)	6,529	411	HS student athletes	Self-report	26.1%^h^	17.1%^h^	<0.001
Nelson et al. (2016) (10)	NR (18.0)	8,056	242	HS & COL athletes	Interview	--^i^	--^i^	<0.001
Gunn et al. (2022) (20)	NR (19.3)^e^	33,387	1,513	COL age	Self-report^j^	35.2%	22.1%	<0.01
Alosco et al. (2014) (4)	NR (NR)	139	14	COL athletes	Self-report	50.4%^k^	14.4%	<0.01
Memmini et al. (2023) (39)	NR (NR)	23,506	1,550	COL athletes	Self-report^j^	10.1%	7.5%	--

Studies are listed in order from youngest mean age to oldest mean age (where mean ages are reported). ADHD, Attention-deficit/hyperactivity disorder. MS, middle school; HS, high school; COL, collegiate; NR, not reported. ^a^The results from Cook, Karr, et al. (12), when stratified by gender, are 7.3% of boys with ADHD versus 6.9% of girls with ADHD (there was no significant difference between the genders). ^b^The results from Iverson et al. (8), when stratified by gender, are 24.5% of boys with ADHD versus 14.3% of boys without ADHD, and 22.2% of girls with ADHD versus 8.1% of girls without ADHD (*p* = 0.051 for the comparison for boys with ADHD and *p* = 0.04 for girls with ADHD). ^c^The results from Cook, Teel, et al. (5), when stratified by gender, are 48.1% of boys with ADHD versus 38.4% of boys without ADHD (*p* = 0.02) and 39.1% of girls with ADHD versus 36.7% of girls without ADHD (*p* = 0.58). ^d^Hrabarchuk et al. (36) reported a lifetime history of 2 or more concussions. ^e^Total sample mean age was not available, so this mean was pooled from the control and ADHD group’s mean ages. ^f^The statistical comparison from Mautner et al. (37) that is presented here is from a chi-square analysis comparing history of 0, 1, and 2+ concussions. ^g^McLeod et al. (11) report frequency in a figure, so these numbers are approximate estimations based on that figure. ^h^The results from Iverson et al. (6), when stratified by gender, are 27.0% of boys with ADHD versus 20.0% of boys without ADHD and 23.6% of girls with ADHD versus 13.6% of girls without ADHD. ^i^Nelson et al. (10) did not report the percentage of the control group with a history of concussion. Rather, they reported frequency by number of lifetime concussions (i.e., 0, 1, 2, or 3+ concussions). They found higher frequency of 1, 2, and 3+ lifetime concussions in those with ADHD (Frequencies of 3.8, 5.9, and 10.6%, respectively). ^j^These studies used data from the CARE Consortium (with slightly different samples for each study). Memmini et al. (39) did exclude military service cadets, while Gunn et al. (20) did not exclude military service members from their sample. ^k^Alosco et al.’s (4) sample consisted of 52% male collision athletes (i.e., football players) and 48% female contact and limited contact athletes (i.e., basketball, soccer, field hockey, and volleyball) which may have influenced the frequency of concussion found in this sample.

By way of brief definition, a prevalence ratio indicates the prevalence of an outcome (e.g., concussion) in one group relative to another group (e.g., those with and without ADHD). For example, among 8- and 9-year-old children from the US general population, the prevalence of parent-reported history of concussion among those with ADHD (7.2%) was greater than the prevalence among those without ADHD (3.2%), resulting in a prevalence ratio of 2.25 (12). In a sample of 11- to 14-year-old student athletes, the prevalence ratio was 2.10 (8). In a sample of high school student athletes, the prevalence ratio was 1.52 (7). In a sample of adolescents seeking specialty concussion care in Montreal, Canada, the prevalence ratio was 1.17 (5). These are select examples, and some of the aforementioned prevalence ratios are reasonably representative of the general population while others are more sample, or subpopulation, specific (i.e., less representative of the general population). Thus, from available studies, the prevalence ratio of lifetime history of concussion based on ADHD status appears to differ slightly by age, with higher prevalence ratios among younger ages. Moreover, the broader ADHD and concussion history literature has tended to focus on older children and adolescents (e.g., 13-17-year-olds), which limits our understanding of whether similar results are present among younger children.

Of note, a few prospective studies have examined whether ADHD is associated with greater risk of sustaining a future concussion (incidence), with conflicting results. Specifically, Chrisman et al. (17) found no association between ADHD and risk for sport-related concussion in a two-year prospective cohort study of 5- to 14-year-old American football players (*n* = 863). Similarly, Cook and Iverson (18) did not find an association between ADHD and risk of concussion in a one-year prospective cohort study of 9 to 10-year old children from the U.S. general population (*n* = 11,013). McLeod et al. (11) found that ADHD was associated with increased incidence of concussion during their two-year study period in high school student athletes (*n* = 8,814). Finally, two studies analyzing data from the CARE consortium (a prospective multi-center study or collegiate athletes and military service members) found that ADHD was associated with a modestly increased risk for concussion during the study timeframe, but only among males and only when an athletes has co-occurring ADHD and LD, although it should be noted that these findings rely on small to very small cell sizes; *n* = 138 with ADHD and 109 with ADHD+psychostimulant use out of a total sample of 4,387 in Coffman et al. and *n* = 1,513 with ADHD and 323 with co-occurring ADHD/LD out of a total sample of 33,387 in Gunn et al. (19, 20).

ADHD is frequently treated with medication (e.g., stimulants) and these medications may reduce the severity of symptoms and functional problems for some individuals (21–23). Taking stimulant medications has been associated with a lower risk for orthopedic injuries (3, 24) and brain injuries of all severities (25–27) among children and adolescents with ADHD. However, research suggests that, in collegiate athletes, those with ADHD who are taking psychostimulants may be slightly more likely to sustain a concussion (although these results did not reach clinical significance) (19). Thus, it is unclear whether youth from the general population with ADHD who are taking medication have a lower rate of concussion than those who are not taking medication. We hypothesized that those youth who were taking ADHD medication would have a lower rate of parent-reported lifetime concussion history compared to those youth with ADHD who were not taking medication. It is also possible that those with more severe forms of ADHD (i.e., more symptoms, greater severity of symptoms, or worse functional impairment) may exhibit higher rates of concussion possibly due to increased functional impact of their hyperactivity, inattention, and/or impulsivity symptoms. Unfortunately, there is a paucity of literature regarding the possible association between ADHD severity and lifetime concussion rates so a data-informed hypothesis could not be formed; however, we hypothesized (based on theory) that more severe parent-reported ADHD would be associated with higher rates of parent-reported concussion history.

Most of the literature on ADHD and concussion history has relied on self-report data for both ADHD status and for concussion history. Although the majority of athletes (greater than 90%) tend to be consistent in their self-report of ADHD and concussion history status over time (28), there is research to suggest that those with ADHD may be somewhat more likely to inconsistently report their ADHD history at two time points compared to those without ADHD (29). While it is not possible to know whether this finding is related to true changes in medical history versus inaccurate reporting, parent-reported diagnosis of ADHD and concussion history may be more accurate and is worthy of study. A recent study of the consistency of parent-reported ADHD diagnosis over time found that 95.7% of parents who reported that their child had ADHD were consistent and indicated that their child had ADHD when asked years later (30). As can be seen in Table 1, only three out of the 15 studies we identified on the topic of ADHD and lifetime concussion history considered parent-reported diagnosis of ADHD, only two used psychiatrist confirmed diagnosis or structured interview, and the remaining 10 studies relied on self-report data from the youth participant, which potentially limits our understanding of the association between ADHD and concussion history.

The current study investigated the association between parent-reported ADHD, ADHD severity, and medication status with lifetime concussion history and whether such associations differ by age in a national sample of children and adolescents ages 5 to 17. We hypothesized that parent-reported history of ADHD would be associated with greater lifetime history of concussion, and that there would be differences in this association across age groups, with the association being stronger among younger children relative to older children and adolescents. We also hypothesized that, among youth with ADHD, those who were taking ADHD medication and those with mild (versus moderate/severe) ADHD would have lower lifetime concussion history.

## 2 Materials and methods

### 2.1 Participants

Participants were drawn from the 2021 National Survey of Children’s Health (NSCH) administered by the U.S. Department of Health and Human Services’ Child and Adolescent Health Measurement Initiative (31). Households across the United States were randomly selected and contacted via mail to invite survey participation. Parents and other caregivers completed the survey by providing information about their child. For simplicity and conciseness, we will refer to parent and caregiver reported data as ‘parent-reported’ throughout. When multiple children resided in the household, one child was randomly selected by the parent or caregiver respondent to be the subject of the data provided in the survey (i.e., the child for which the parent or caregiver was providing health information). Survey questions related to, among other areas, the child’s health status, demographics, mental health difficulties, neurodevelopmental history (including ADHD), and activities. From the overall NSCH dataset, participants were selected for the current study if they had complete data on the primary concussion and ADHD questions.

The 2021 NSCH dataset included 50,892 cases, of which 16,672 were less than 5 years of age and were not included in this study—leaving 34,220 subjects between the ages of 5 and 17. The following exclusions were made in the following order: missing data on the concussion history variable (*n* = 143) and missing data for lifetime ADHD status (*n* = 163). The final sample consisted of parent-reported data for 33,914 children and adolescents (ages 5 to 17; mean = 10.98 years, *SD* = 3.98). Girls represented 47.9% of the sample. The parent-reported racial identities of youth in the sample were as follows: White (76.4%), Black/African American (7.4%), biracial/multiracial (8.4%), Asian (5.9%), American Indian/Alaska Native (1.1%), and Native Hawaiian/Other Pacific Islander (0.8%). The ethnic composition of the sample was as follows: Hispanic or Latino Origin (13.7%) and not of Hispanic or Latino Origin (86.3%).

### 2.2 Variables of interest

The exact variable names from the NSCH database for the variables of interest are presented in Table 2. Brief descriptions of the variables are provided below.

**Table 2:** Study variables of interest in the 2021 NSCH database.

Variable description	Variable name in 2021 NSCH data	Precise wording of the question in the survey
Concussion history	CONCUSSION	Do you think this child has ever had a concussion or brain injury? A concussion or brain injury is when a blow or jolt to the head causes problems such as headaches, dizziness, being dazed or confused, difficulty remembering or concentrating, vomiting, blurred vision, changes in mood or behavior, or being knocked out.
ADHD History	K2Q31A	Has a doctor or other health care provider EVER told you that this child has…Attention Deficit Disorder or Attention-Deficit/Hyperactivity Disorder, that is, ADD or ADHD?
Current ADHD	K2Q31B	If [K2Q31A is] yes, does this child CURRENTLY have the condition?
ADHD Severity	K2Q31C	If [K2Q31B is] yes, is it: Mild, Moderate, or Severe.
ADHD Medication	K2Q31D	[If K2Q31A is yes] Is this child CURRENTLY taking medication for ADD or ADHD?

ADHD, attention-deficit/hyperactivity disorder; NSCH, National Survey of Children’s Health.

#### 2.2.1 Concussion and traumatic brain injury (TBI) history

For lifetime concussion history, parents responded to the following question: “Do you think this child has EVER had a concussion or brain injury? A concussion or brain injury is when a blow or jolt to the head causes problems such as headaches, dizziness, being dazed or confused, difficulty remembering or concentrating, vomiting, blurred vision, changes in mood or behavior, or being knocked out.” Parents responded “yes” or “no” to this question. For simplicity and ease of readability, we will refer to this variable as ‘lifetime history of concussion’ throughout.

#### 2.2.2 ADHD status

The primary ADHD question in the survey asks: “Has a doctor or other health care provider EVER told you that this child has…Attention Deficit Disorder or Attention-Deficit/Hyperactivity Disorder, that is, ADD or ADHD?” A total of 4,560 parents reported that they had been told by a health care provider that their child had ADHD. Parents who responded “yes” to this lifetime history of ADHD question were then asked to clarify if their child *currently* has the condition.

#### 2.2.3 ADHD severity

Parents who reported that their child currently has ADHD were asked follow-up questions, including the severity of their child’s ADHD: “If yes, is it: Mild, Moderate, or Severe.” While it is not specified in the question stem, we believe that the severity variable likely relates to current rating of severity (given the previous question regarding current status of ADHD, as opposed to lifetime history of ADHD which is a different variable in this dataset). The moderate and severe groups were combined, forming two severity subgroups: (i) mild and (ii) moderate or severe. There were 23 missing cases on the ADHD severity variable (0.6% of the 4,161 individuals with parent-reported ADHD diagnosis).

#### 2.2.4 ADHD medication status

The medication status question asked parents to clarify their child’s current ADHD medication status. Specifically, “Is this child CURRENTLY taking medication for ADD or ADHD?.” There were 20 missing cases on the ADHD medication status variable (0.5% of the 4,161 individuals with parent-reported ADHD diagnosis).

### 2.3 Statistical analyses

Participants were separated into five age groups based on developmental similarity: 5–7-year-olds, 8-10-year-olds, 11-13-year-olds, 14-15-year-olds, and 16-17-year-olds. Mantel–Haenszel analysis is a statistical technique that conducts two-by-two chi-square analyses (with dichotomous outcome and predictor variables) stratified by two or more levels of a confounding factor (e.g., age groups, ADHD severity levels, etc.). In the current study, a Mantel–Haenszel analysis was used to compare the associations between parent-reported ADHD and lifetime concussion history across the five age groups. Additional Mantel–Haenszel analyses were used to compare the associations between lifetime history of concussion and parent-reported ADHD severity as well as ADHD medication status across the different age groups. The initial Mantel–Haenszel test was an omnibus test, that included the entire sample and statistically compared all age bands simultaneously. We then conducted post-hoc analyses between the age bands. Prevalence ratios indicate the prevalence of an outcome (e.g., concussion) in one group relative to another group (e.g., those with and without ADHD). Prevalence differences indicate the number of excess cases of a condition (e.g., concussion) that can be expected in one group versus another (e.g., those with and without ADHD). Prevalence ratios and prevalence differences were calculated for each of the age groupings comparing the prevalence of lifetime concussion between children or adolescents with ADHD to those without ADHD. We attempted to stratify our primary analyses by sex and ADHD status across the age groups; however, we encountered small and very small cell sizes in the younger age groups and so further analysis was not possible.

## 3 Results

A total of 4,161 parents reported that their child had a current diagnosis of ADHD. The other 399 parents denied current ADHD and were excluded. Thus, the final sample of those with ADHD included the 4,161 parents who reported their child had been diagnosed with ADHD and that their child currently carried the diagnosis. In the total sample, 6.2% of youth had a parent-reported lifetime history of concussion (*n* = 2,103) and 12.3% had a history of parent-reported ADHD (*n* = 4,161). The number of children and adolescents with and without parent-reported lifetime concussion history is presented in Table 3. In the total sample, boys had a slightly greater parent-reported lifetime concussion history compared to girls (7.1% versus 5.2% of girls; χ^2^ = 56.60; *p* < 0.001; odds ratio (OR) = 1.41; 95% CI for OR = 1.29–1.54). Boys were also more likely to have a history of ADHD (15.7% versus 8.6% of girls; χ^2^ = 396.87; *p* < 0.001; OR = 1.98; 95% CI for OR = 1.85–2.12). In the total sample, the proportion of children and adolescents with parent-reported history of concussion increased with age (2.2% of 5-7-year-olds compared to 12.4% of 16-17-year-olds; see Table 3).

**Table 3:** Parent-reported lifetime history of concussion in children and adolescents by ADHD status.

Variable	Total Sample			ADHD			No ADHD		
	*n*	*f*	%	*n*	*f*	%	*n*	*f*	%
Total sample	33,914	2,103	6.2%	4,161	441	10.6%	29,753	1,662	5.6%
Age groups									
Ages 5–7	8,833	193	2.2%	479	28	5.8%	8,354	165	2.0%
Ages 8–10	6,782	272	4.0%	889	56	6.3%	5,893	216	3.7%
Ages 11–13	6,998	401	5.7%	1,085	105	9.7%	5,913	296	5.0%
Ages 14–15	5,322	497	9.3%	807	108	13.4%	4,515	389	8.6%
Ages 16–17	5,979	740	12.4%	901	144	16.0%	5,078	596	11.7%
Sex									
Girls	16,238	840	5.2%	1,391	148	10.6%	14,847	692	4.7%
Boys	17,676	1,263	7.1%	2,770	293	10.3%	14,906	970	6.5%

ADHD, attention-deficit/hyperactivity disorder; n = sample size; *f* = frequency.

### 3.1 Lifetime concussion history among those with and without ADHD across age and gender

**Figure 1:**
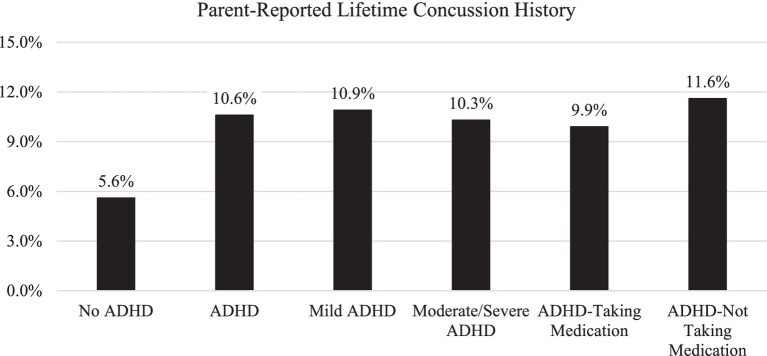
Lifetime history of parent-reported concussion by ADHD diagnosis, severity, and medication status. ADHD, attention-deficit/hyperactivity disorder. Total sample size *n* = 33,914. ADHD *n* = 4,161. No ADHD *n* = 29,753. Mild ADHD *n* = 1,654. Moderate/Severe ADHD *n* = 2,484. ADHD- Taking Medication group *n* = 2,472. ADHD-not taking medication group *n* = 1,669.

**Figure 2:**
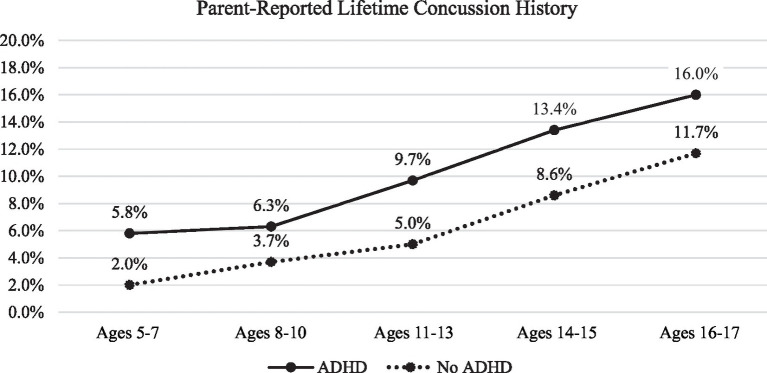
Parent-reported concussion history by age band and ADHD status. ADHD, attention-deficit/hyperactivity disorder. Sample sizes for these subgroups are reported in Table 3.

The frequencies of parent-reported lifetime history of concussion by age band, ADHD status, and gender are presented in Table 3. The results of the Mantel–Haenszel comparisons by ADHD status, ADHD severity, and medication status are presented in Table 4. The percentages of those with a lifetime history of concussion by ADHD group for the overall sample are presented in Figure 1. The percentages of those with a lifetime history of concussion by age group (e.g., 5-7-year-olds versus 8-10-year-olds, etc.) are presented in Figure 2.

**Table 4:** Mantel–Haenszel comparisons of parent-reported concussion history by ADHD status, ADHD severity, and medication status across age groups.

Age groups	Concussion history	ADHD status					ADHD severity					Medication status				
		ADHD	No ADHD	χ^2^	OR	95% CI	Mild	Mod/Sev	χ^2^	OR	95% CI	Meds	No Meds	χ^2^	OR	95% CI
Ages 5–7	Yes	28	165	31.75*	3.08	2.04–4.65	10	17	0.31	0.80	0.36–1.78	13	15	0.21	0.84	0.39–1.80
	No	451	8,189				143	305				227	219			
Ages 8–10	Yes	56	216	13.92*	1.77	1.31–2.39	24	31	1.69	0.70	0.40–1.21	31	25	2.03	0.67	0.39–1.16
	No	833	5,677				290	539				537	292			
Ages 11–13	Yes	105	296	37.04*	2.03	1.61–2.57	33	71	2.36	1.40	0.91–2.16	64	39	0.16	0.92	0.60–1.40
	No	980	5,617				385	591				628	351			
Ages 14–15	Yes	108	389	18.38*	1.64	1.31–2.06	44	64	0.72	1.20	0.79–1.80	60	48	0.56	0.86	0.57–1.29
	No	699	4,126				313	381				412	282			
Ages 16–17	Yes	144	596	12.72*	1.43	1.17–1.74	70	74	0.50	0.88	0.62–1.26	77	67	0.34	0.90	0.63–1.28
	No	757	4,482				342	411				423	331			
Mantel-Haenszel OR	--	--	--	91.86*	N/A	N/A	--	--	0.02	0.94	0.77–1.15	--	--	2.19	0.84	0.69–1.02

OR, odds ratio. 95% CI, 95% confidence interval for the odds ratio. ADHD, attention-deficit/hyperactivity disorder. Mod/Sev, Moderate/Severe. Meds, On ADHD medication. No Meds, Not on ADHD medication. N/A, not applicable because of significant Breslow-Day test suggesting heterogeneity in the odds ratios that would preclude interpretation of the common odds ratio. ^*^p < 0.001.

Youth with ADHD (10.6%) were more likely than those without ADHD (5.6%) to have a parent-reported history of concussion (prevalence ratio = 1.9; prevalence difference = 5.0 per 100). This was the same for boys and girls with ADHD (10.3% of boys with ADHD had a concussion history and 10.6% of girls with ADHD had a concussion history). Youth with ADHD were more likely to have a history of concussion across all 5 age bands (*χ^2^_Mantel–Haenszel_* = 91.86, *p* < 0.001). Odds ratios differed across age-groups (*χ^2^_Breslow-Day_* = 13.46, *p* = 0.009). The greatest difference (largest effect size) was found in the 5-7-year-old age group where 5.8% of those with ADHD had a history of concussion compared to 2.0% of those without ADHD (prevalence ratio = 3.0; prevalence difference = 3.9 per 100; odds ratio of 3.08). Differences between the other age bands were as follows: 8-10-year-old odds ratio = 1.77, prevalence ratio = 1.7; prevalence difference = 2.6 per 100; 11-13-year-old odds ratio = 2.03, prevalence ratio = 1.9, prevalence difference = 4.7 per 100; 14-15-year-old odds ratio = 1.64, prevalence ratio = 1.6, prevalence difference = 4.8 per 100; and 16-17-year-old odds ratio = 1.43, prevalence ratio = 1.4, prevalence difference 4.2 per 100. As can be seen in Figure 2, although the odds ratios and prevalence ratios differed by age group, the absolute difference between youth with and without ADHD is small and is maintained at each age band. In general, those with ADHD had around 4.0% higher frequency of concussion compared to those without ADHD (absolute frequency differences among the age bands ranged from 2.6 to 4.8%). Moreover, the prevalence differences indicate similarity in the absolute differences across age groupings, ranging from 2.6 to 4.8 excess concussion cases per 100 among youth with ADHD compared to youth without ADHD.

### 3.2 ADHD severity and medication status

The frequencies of parent-reported lifetime history of concussion by age band, ADHD severity, and ADHD medication status are presented in Table 5. There were no significant differences in lifetime concussion history for those with mild ADHD versus moderate/severe ADHD (*χ^2^_Mantel–Haenszel_* = 0.02, *p* = 0.90). Similarly, there were no differences in lifetime concussion history for those with ADHD taking medication versus those with ADHD who were not taking medication (*χ^2^_Mantel–Haenszel_* = 2.19, *p* = 0.14). Children/adolescents with moderate/severe ADHD were more likely to be on medication compared to those with mild ADHD (*χ^2^* = 246.46, *p* < 0.001). Specifically, 69.5% of those with moderate/severe ADHD were reportedly on medication versus 45.0% of those with mild ADHD. The lifetime concussion history status of those with mild versus moderate/severe ADHD and those on medication versus no medication is presented in Figure 1.

**Table 5:** Parent-reported lifetime history of concussion in children and adolescents by ADHD severity and medication status.

Age range	ADHD severity					ADHD medication status				
	Mild		Moderate/Severe		*p*	Medication		No medication		*p*
	*f*	%	*f*	%		*f*	%	*f*	%	
Ages 5–7	10	6.5%	17	5.3%	0.58	13	5.4%	15	6.4%	0.65
Ages 8–10	24	7.6%	31	5.4%	0.19	31	5.5%	25	7.9%	0.16
Ages 11–13	33	7.9%	71	10.7%	0.13	64	9.2%	39	10.0%	0.69
Ages 14–15	44	12.3%	64	14.4%	0.40	60	12.7%	48	14.5%	0.45
Ages 16–17	70	17.0%	74	15.3%	0.48	77	15.4%	67	16.8%	0.56
All Ages	181	10.9%	257	10.3%	0.54	245	9.9%	194	11.6%	0.08

ADHD, attention-deficit/hyperactivity disorder. *f* = frequency of parent-reported lifetime history of concussion. There was missing data on ADHD severity for 23 participants. There was missing data for ADHD Medication Status for 20 participants.

## 4 Discussion

In this national sample of children and adolescents from across the United States, youth with ADHD had twice the prevalence of parent-reported lifetime history of concussion compared to youth without ADHD (10.6% compared to 5.6%). This finding is consistent with many prior studies showing that children and adolescents with ADHD are more likely to report a lifetime history of concussion. The present study is novel in that this dataset included a broad age range (5 to 17), allowing us to examine whether the magnitude of this well-established association between ADHD and greater lifetime history of concussion is consistent or whether it differs by age. While our results indicate that the odds ratios (effect sizes) differ across age bands (e.g., OR of 3.08 in 5-7-year-olds versus 1.77 in 8-10-year-olds, etc.), the absolute difference in the percentage of youth with and without ADHD who have a history of concussion is relatively small and stable across these ages. As can be seen in Figure 2, those with ADHD had, on average, a 4% higher frequency of parent-reported lifetime concussion history compared to those without ADHD, and this absolute difference is maintained at each age band.

These results, represented in prevalence ratios, are consistent with prior studies with samples with narrower age ranges, including 8–9 year olds from the U.S. general population (12), 11–14 year old middle school student athletes from Virginia (8), USA, and 13–18 year old high school student athletes from Maine, USA (7). Specifically, the estimated magnitudes of difference in lifetime prevalence of concussion (prevalence ratios) in the current sample are consistent with those from prior studies. The prevalence ratio (PR) for youth ages 8–10 years in the current study (PR = 1.7) is similar to, though slightly lower than a previously reported national sample of youth ages 8 to 9 years old (PR = 2.3) (12). For youth ages 11 to 13 in the current study, the PR of 1.9 is comparable to results from middle school student athletes in Virginia (PR = 2.10) (8). And for adolescents ages 14 to 15 and 16 to 17, PRs in the current sample (PR = 1.6 and PR = 1.4, respectively) are comparable to a study of high school student athletes from Maine (PR = 1.5) (7).

Taken together, these findings suggest that the increased risk of concussion for youth with ADHD, at least those in the general population (as opposed to youth athletes), occurs by age 7, with that early difference then maintained over time. If this finding is replicated, ideally with prospective studies, it might suggest the need for developmentally targeted injury prevention strategies for young children who are diagnosed with or who are considered at-risk for ADHD. Additionally, it is unclear if these findings are consistent with youth athletes (as opposed to a population-based cohort of children and adolescents) and so further investigation in athlete populations may be warranted. Future work should also compare risk for concussion and risk for other types of physical injury among young children with ADHD, as the difference noted in our study might represent a more general increased risk for bodily injury associated with ADHD, as opposed to a unique vulnerability to concussion per se (i.e., an increased risk for any type of injury rather than a potential increased neurophysiological susceptibility to concussion).

Contrary to our hypotheses, there were no differences in history of concussion based on parent-reported ADHD severity or ADHD medication status. It is important to appreciate that the severity ratings were made by parents and not clinicians. Per the Diagnostic and Statistical Manual, 5^th^ Edition, Text Revision (DSM-5-TR) (1), ADHD severity levels are based on such factors as the number of symptoms and the extent of functional impairment. For example, a specifier of ‘mild’ ADHD refers to an individual experiencing few symptoms in excess of those required for diagnosis and minor functional impairment in daily living whereas ‘severe’ ADHD is characterized by many excess symptoms and marked functional impairment. This severity rating would typically be applied by clinicians after thorough evaluations of children, including assessment of their functioning in different domains such as in a school setting versus in a home setting. In the current study, on the other hand, parents reported how severe they perceived their child’s symptoms were by responding to a single question with no anchors or specification for what ‘mild’ versus ‘severe’ refers to. It is possible that future studies using clinician confirmed diagnoses and clinician-assigned severity ratings may come to different conclusions than those found here.

Regarding ADHD medication status, we found a slightly lower rate of lifetime concussion history in youth taking ADHD medication; however, these results were not statistically significant (*p* = 0.08). The results are somewhat contrary to prior literature. Specifically, previous research has found that children, adolescents, and young adults with ADHD who are taking psychostimulant medication have lower rates of TBI (of all severity levels) and unintentional orthopedic injuries (25–27, 32, 33). Similar results are found in studies investigating concussion (rather than TBI broadly) in youth athlete and non-athlete populations (19, 33, 34). In the current study, medication use was parent-reported for current use; however, there was no way to analyze the temporal association between medication use and concussion. That is, it was unknown whether youth in the current study were actively prescribed or taking medication at the same time as their concussion. In one national cohort study (*N* = 2,319,450), the rate of unintentional injury via motor vehicle accidents in individuals with ADHD was reduced in those using psychostimulant medication on a long-term basis (2 years). In that study long-term medication users had 22% fewer accidents than those who were not taking medication on a long-term basis. This association remained significant even after accounting for current medication use at the time of injury suggesting that longer-term treatment may have a protective effect above and beyond current medication usage. Given this, and the limitations of the medication variable in the current study (see limitations section below), we are unable to clearly hypothesize how our current findings may relate to ADHD medication use and concussion risk. We also found that those with moderate/severe ADHD were also more likely to be taking medication (*p* < 0.001; with 69.5% of youth with moderate/severe ADHD reportedly taking medication versus 45.0% of those with mild ADHD), which may further confound our findings.

The current study has several strengths and weaknesses. First, our data were selected from a nationally representative survey of children and adolescents in the United States, which increases the generalizability of our results. Additionally, our data focused on parent-reported ADHD status and concussion history. As can be seen in Table 1, the vast majority of the literature in this area has examined youth self-reported concussion history and ADHD status. Inclusion of parent-reported data can be helpful to clarify the association between ADHD status and concussion history by providing another perspective on the youth’s history. It is possible, and at some ages very likely, that a child or adolescent might not know whether or not they have been diagnosed with ADHD, but their parents would be expected to know this information quite well. Additionally, our study included a broad age range (ages 5 to 17). Prior literature (see Table 1) has tended to include adolescent samples and/or samples with limited age ranges (e.g., 9 to 10 year olds only, 12 to 13 year olds only). By including a broader range of ages in the current study, we were able to assess for possible differences in associations between ADHD and concussion history in younger versus older children and adolescents. This study is particularly useful because it adds to the literature in younger children with ADHD (i.e., youth under age 7), which have not often been a focus of study on this topic.

Limitations of the current study include the self-report nature of the data as well as limitations in the available variables measuring both ADHD and concussion (dichotomous measures of presence and absence, as opposed to continuous measures, such as the number of ADHD symptoms endorsed or the number of lifetime concussions sustained). Further, the exact ADHD medication a child was taking was not reported in the dataset, nor was the dosage; additional information about the specific medication might have allowed for analyses to be conducted comparing classes of medication (e.g., psychostimulant versus non-stimulant medication). This might represent an area of future study. Additionally, parent-reported severity of ADHD symptoms in this particular dataset represented the adult caregivers’ perceptions of ADHD symptomatology with no behavioral anchors for a mild versus severe problem provided in the question stem of the item in the survey. It is also possible that parents reported current levels of severity (as opposed to severity of the disorder at its onset). This could be further confounded by the possibility that those with initially severe symptoms may be more likely to be placed on ADHD medication, which may, in turn, lead to lower severity of symptoms. Operationalizing ADHD severity by number of symptoms, level of functional impairment, and/or providing behavioral anchor examples would serve to better represent the construct of severity in future studies. Additionally, lack of information about how parent-reported concussions were sustained (such as through sporting contexts versus falls), the cross-sectional nature of the data, and reliance on a single reporter (the parent/guardian) are also weaknesses. As can be said for most of the research in this area, the use of clinician confirmed diagnosis of ADHD would represent an aspirational standard for establishing ADHD status compared to youth self-report or even parent-report of ADHD status. While self-report and parent-reported data can be beneficial and can help to enhance our understanding of this topic, future research should aim to identify ADHD status using clinician confirmed diagnostic methods.

## 5 Conclusion

In this national sample from the United States, youth with ADHD had twice the rate of lifetime concussion history than youth without ADHD. This finding was present even in young children (i.e., 5- to 7-year-olds) with the largest effect size found in these younger children compared to older children and adolescents. The absolute difference in concussion history for those with and without ADHD, however, was relatively small and stable across age bands (see Figure 2). In those youth with ADHD, there was no association between parent-reported severity of ADHD or medication status and lifetime history of concussion. The latter findings are limited, however, given that ADHD severity was parent-reported (general parental perception of severity) as opposed to being determined by formal clinical assessment, symptom count, and/or functional difficulties. Additionally, there was no available data regarding the specific ADHD medication, the youth’s medication dosage, or the temporal relationship between medication use and concussion injury.

## Data Availability

The original contributions presented in the study are included in the article, further inquiries can be directed to the corresponding author. This study uses public health data derived from the National Survey of Children’s Health (NSCH), conducted by the US Census Bureau in 2021. Child and Adolescent Health Measurement Initiative (CAHMI) [2021]. 2021 National Survey of Children’s Health, SPSS Indicator dataset. Data Resource Center for Child and Adolescent Health supported by Cooperative Agreement from the US Department of Health and Human Services, Health Resources and Services Administration (HRSA), Maternal and Child Health Bureau (MCHB). Retrieved 09/17/23 from www.childhealthdata.org.
